# A Comparative Study on Permanent Mold Cast and Powder Thixoforming 6061 Aluminum Alloy and Sic_p_/6061Al Composite: Microstructures and Mechanical Properties

**DOI:** 10.3390/ma9060407

**Published:** 2016-05-24

**Authors:** Xuezheng Zhang, Tijun Chen, He Qin, Chong Wang

**Affiliations:** State Key Laboratory of Advanced Processing and Recycling of Nonferrous Metals, Lanzhou University of Technology, Lanzhou 730050, China; zhangxz1991@163.com (X.Z.); qinhe19900927@126.com (H.Q.); wangchong19890329@126.com (C.W.)

**Keywords:** powder thixoforming, permanent mould cast, SiC_p_/6061Al composite, microstructure, tensile properties, strengthening mechanisms

## Abstract

Microstructural and mechanical characterization of 10 vol% SiC particles (SiC_p_) reinforced 6061 Al-based composite fabricated by powder thixoforming (PTF) was investigated in comparison with the PTF and permanent mold cast (PMC) 6061 monolithic alloys. The results reveal that the microstructure of the PMC alloy consists of coarse and equiaxed α dendrites and interdendritic net-like eutectic phases. However, the microstructure of the PTF composite, similar to that of the PTF alloy, consists of near-spheroidal primary particles and intergranular secondarily solidified structures except SiC_p_, which are distributed in the secondarily solidified structures. The eutectics amount in the PTF materials is distinctly lower than that in the PMC alloy, and the microstructures of the former materials are quite compact while that of the latter alloy is porous. Therefore, the PTF alloy shows better tensile properties than the PMC alloy. Owing to the existence of the SiC reinforcing particles, the PTF composite attains an ultimate tensile strength and yield strength of 230 MPa and 128 MPa, representing an enhancement of 27.8% and 29.3% than those (180 MPa and 99 MPa) of the PTF alloy. A modified model based on three strengthening mechanisms was proposed to calculate the yield strength of the PTF composite. The obtained theoretical results were quite consistent with the experimental data.

## 1. Introduction

Developing low density, high specific stiffness and super dimensional stability materials have always attracted considerable attention for aerospace and automobile industrial applications [1,2]. Aluminum matrix composites (AMCs) reinforced with ceramic particles are particularly attractive because of their superior strength, stiffness and wear resistance. In addition, they have excellent isotropic mechanical and physical properties both at ambient and elevated temperatures in comparison with the conventional monolithic alloys [1]. One main problem still always plagues the incorporation of micro-size ceramic particles into the aluminum matrix: the high clustering tendency of micro-particles [3]. Numerous methods have been developed to overcome this problem such as melt stirring [2,3], ultrasonic dispersion [4], squeeze casting [5,6] and powder metallurgy [7,8]. Among the various processing techniques, powder metallurgy has prominent advantages such as flexible design of the constituents and uniform distribution of ceramic particles [8], but the as-obtained composites inevitably comprise numerous voids, though some advanced sintering techniques are employed. Besides, it is not suitable to apply powder metallurgy to produce parts with large sizes or complex shapes [7]. Thixoforming, a promising metal-forming technology that can markedly diminish voids, has been introduced to be integrated with powder metallurgy and thus a novel technology called PTF is developed. The PTF not only retains the advantages of powder metallurgy, but also inherits the merits of compact microstructure and being available for producing large-sized and complicated components of the thixoforming. Unfortunately, the focus of the existing research into PTF was mainly on the microstructure evolution during partial remelting and mechanical properties of aluminum matrix alloys [9,10,11]. Only a few studies involved composites fabricated by PTF, and they concentrated on the effects brought out by solution treatment [12] or failed to consider the corresponding mechanical properties [13].

The main purpose of this study is to highlight the advantage of this novel technology and elucidate the strengthening mechanisms of SiC_p_ in the PTF-SiC_p_/6061Al composite. To achieve this goal, several 10 vol% of 6.94 µm SiC_p_ reinforced 6061 Al-based composite samples were prepared using PTF, and the resulting microstructure, mechanical properties and fracture behavior were investigated in detail in comparison with those of the matrix alloys fabricated by PMC and PTF, respectively. A modified model on the basis of three strengthening mechanisms was proposed to calculate the yield strength of this composite. The obtained theoretical results were found to be quite consistent with the experimental data.

## 2. Materials and Methods

### 2.1. Fabrication PMC of 6061 Alloy

**Figure 1:**
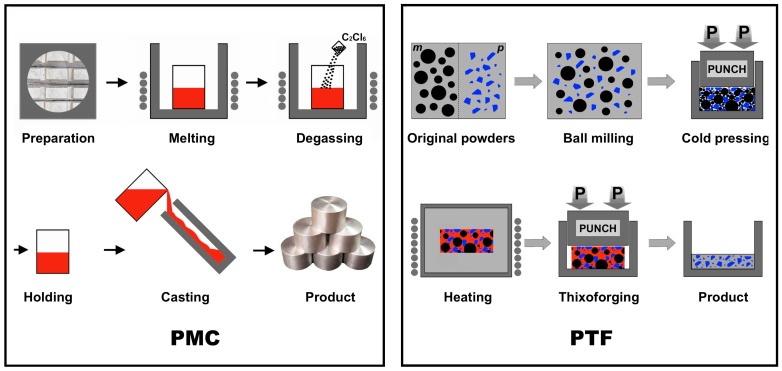
Schematic illustration of the PMC and PTF process (*m*: matrix; *p*: SiC_p_).

The PMC-6061 alloy employed in this work was prepared by pure Al, Mg, Cu and Al-20Si master alloy (all of them were provided by Changfeng factory, Lanzhou, China) and the resulting alloy possessed a nominal composition of Al-1.0Mg-0.6Si-0.3Cu (wt%). A schematic illustration of the PMC process is presented in Figure 1. First, a quantity of the master alloy and pure metals was melted in a resistance furnace at 1023 K. Degassing was then conducted at 1003 K by adding 1.5 wt% C_2_Cl_6_ (Kejia Chemical Company, Ningbo, China) into the melt. The melt was held for 5 min and ultimately injected into a permanent mold (Changfeng factory, Lanzhou, China) with diameters of 50 mm and lengths of 500 mm. Some specimen cakes with diameters of 50 mm and lengths of 15 mm were cut from the cast rods.

### 2.2. Fabrication PTF of Sicp/6061 Composite and 6061 Alloy

The raw materials used in the fabrication of SiC_p_/6061 composite and 6061 alloy were atomized 6061 alloy powders and pre-oxidized SiC particles with an average size of 17.91 µm and 6.94 µm, respectively. For comparison, the schematic diagram of the PTF process for SiC_p_/6061 composite is also presented in Figure 1. First, 6061 alloy powders were mixed with 10 vol% SiC_p_ and the resulting powders were subjected to ball milling in an ND7-21 planetary ball-milling machine (Nanjing Laibu Technology Industry Co., Ltd., Nanjing, China) at a mixing time, rotation speed and ball-to-powder weight ratio of 40 min, 100 rpm and 5:1, respectively. The resultant powder mixture was then cold pressed into a specimen with 50 mm in diameter and 15 mm thickness under a pressure of 150 MPa. Based on the results of an existing research [14], one specimen was heated in a resistance furnace at 933 K for 90 min. Then the heated specimen was quickly put into a forging mold (Changfeng factory, Lanzhou, China) with a cavity 55 mm in diameter and 45 mm deep, and finally thixoformed under an optimized pressure of 160 MPa. Repeating the above experimental procedure, several PTF-SiC_p_/6061Al composite cakes can be obtained. The fabrication process of the PTF-6061 alloy was similar to that of the PTF-SiC_p_/6061Al composite with the exception of ball milling.

### 2.3. Material Characterization

Metallographic specimens 10 mm in diameter and 10 mm long were machined from the central region of a cake specimen, and then ground, finished, polished and etched using an aqueous solution of 10 vol% NaOH (Kejia Chemical Company, Ningbo, China). Subsequently, the specimens were observed on a MeF3 optical microscope (OM; Nikon Instruments, Shanghai, China) and a QUANTA FEG 450 scanning electron microscope (SEM; FEI, Hillsboro, TX, USA) equipped with an energy dispersive spectroscope (EDS). The estimation of the shape factor and volume fraction of the primary particles in the PTF materials as well as the grain size of these three materials were carried out by Image-Pro Plus 6.0 software (Media Cybernetics Company, Silver Spring, MD, USA) utilizing the OM images randomly captured in minimum ten different locations for each specimen. Similar methods were also used to calculate the eutectics fraction of these three materials. The area ratio of the eutectic phases to the whole ones was taken as the eutectics fraction. In attempt to verify the detailed fracture mechanisms of these materials, some typical fracture surfaces and their corresponding side views were observed on the SEM and OM. The density of the specimens was measured by the Archimedes method so as to calculate the porosity of the specimens.

### 2.4 Tensile Testing

The mechanical properties of these three materials were evaluated by tensile testing conducted at room temperature (about 293 K) on a WDW-100D universal material testing machine (Jinan HengXu Testing Machine Technology Co., Ltd., Jinan, China) with a loading velocity of 5 × 10^−3^ m/s. The tensile bars with a gauge length of 10 mm and a cross-section of 2.5 mm long and 1.5 mm wide were cut from the central part of a specimen cake. The average of minimum five tests was taken as the tensile properties of each material.

## 3. Results and Discussion

### 3.1. Microstructural Analysis

**Figure 2:**
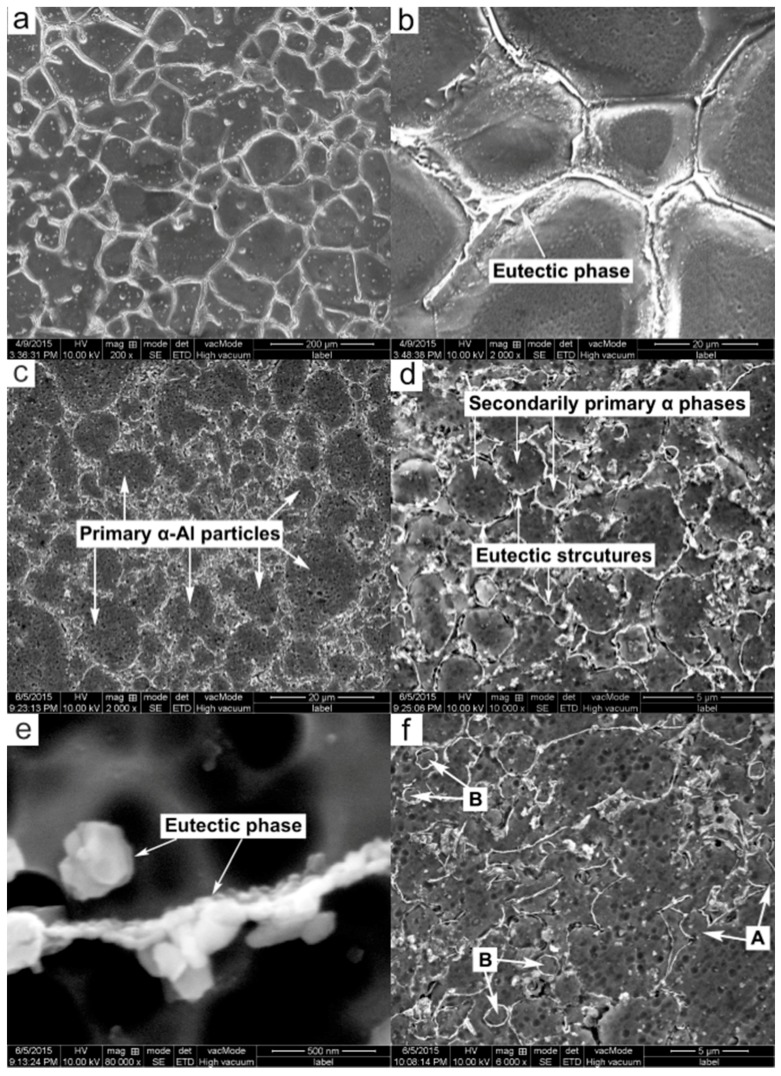
SEM images of: (**a**,**b**) PMC-6061 alloy; (**c**–**f**) PTF-6061 alloy.

Figure 2 shows the microstructures of the PMC-6061 alloy and PTF-6061 alloy. The microstructure of the PMC-6061 alloy is mainly composed of coarse and equiaxed α dendrites with an average size of 90.01 μm and interdendritic net-like eutectic phases (Figure 2a). The eutectic phases of α-Al, Mg_2_Si and Si [15] are distributed in a skeleton shape along the grain boundaries (Figure 2b). As shown in Figure 2c, the microstructure of the PTF-6061 alloy consists of near-spheroidal primary α-Al particles with an average size of only 16.83 μm and intergranular secondarily solidified structures. The secondarily solidified structures are comprised of small spheroidal secondarily primary α grains (to differentiate from the primary α-Al particles, the primary α grains solidified from the liquid phase in a semi-solid state are named as secondarily primary α grains) and intergranular eutectic structures (Figure 2d). Similar to the PMC alloy, the eutectic phases are also distributed in skeleton shapes along the grain boundaries (Figure 2e). Based on the phase constituents and microstructure of the PTF alloy as discussed above, the secondarily solidification process can be divided into three stages. First, due to the presence of the primary α-Al particles, the secondarily primary α-Al particles grow directly on the surfaces of the primary particles without nucleation [16], resulting in the formation of annular or serrated structure attachments to the primary α-Al particles (marked by arrows A in Figure 2f). Then, nucleation appears in the liquid phase far away from the primary particles, resulting in the generation of numerous small globular α grains (marked by arrows B in Figure 2f). Finally, the solidification is completed by the eutectic reaction of L *→* α-Al + Mg_2_Si + Si. Overall, the microstructures under these two forming techniques are distinctly different. The microstructure of the PMC-6061 alloy is composed of coarse and equiaxed α dendrites with an average size of 90.01 μm and interdendritic net-like eutectic phases, while that of the PTF-6061 alloy consists of near-spheroidal primary α-Al particles with an average size of only 16.83 μm and intergranular secondarily solidified structures.

**Figure 3:**
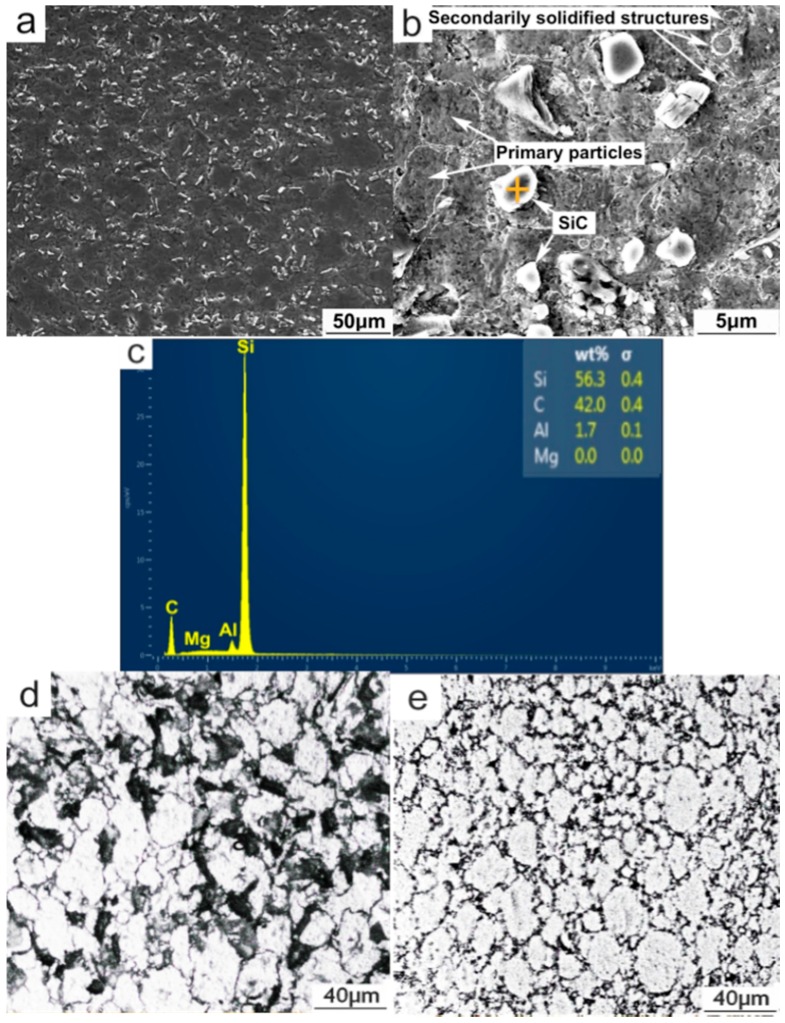
(**a**,**b**) SEM images of PTF-SiCp/6061Al composite; (**c**) EDS result of the cross in Figure 3b proving the presence of SiCp; (**d**) OM micrograph of PTF-SiCp/6061Al composite; (**e**) OM micrograph of PTF-6061 alloy.

For the PTF-SiC_p_/6061 Al composite, SiC reinforcements are uniformly distributed in the matrix under the low-magnification SEM image (Figure 3a). The microstructure, similar to that of the PTF-6061 alloy, also consists of near-spheroidal primary particles and intergranular secondarily solidified structures except the SiC_p_, which distribute in the secondarily solidified structures (Figure 3b,c). The statistics indicate that the shape factor and volume fraction of the primary particles in the PTF composite attain values of 2.3% and 89.3%, respectively (Figure 3d), which are higher than those (1.8% and 87.6%) in the PTF alloy (Figure 3e), disclosing a more irregular morphology of the primary particles (if the particles are perfectly spherical, the shape factor has a value of 1; it increases for less spherical particles [17]) and fewer secondarily solidified structures in the PTF composite. Both of the PTF materials were reheated at the same temperature (933 K) during partial remelting. However, the existence of SiC_p_ would impede the heat transfer from the edge to the center of the semisolid ingot due to its low thermal conductivity and the microstructural evolution rate of the PTF composite is thereby delayed. This accounts for the higher volume fraction of primary particles in the PTF composite. In accordance with a report by Chen *et al.* [11], the microstructural evolution of 6061 alloy powders during partial remelting was divided into three stages:

- the initial coarsening
- structure separation and spheroidization
- final coarsening

It can be expected that the spheroidization stage of the PTF composite should lag behind that of the PTF alloy owing to the delayed heat transferring in the former material. That is, when all of the primary particles in the PTF alloy were fully spheroidized and entered into the final stage, the spheroidization stage in the PTF composite may just occur because of its delayed evolution rate. Therefore, the primary particles of the PTF composite are less spherical, *i.e.*, its shape factor is higher than that of the PTF alloy. The quantitative examination also indicates that the average size (16.51 µm) of the primary particles of the PTF composite is slightly smaller than that of the PTF alloy (comparing Figure 3d,e). With respect to the SiC_p_, two main factors influence the semisolid microstructure prior to thixoforming. First, as mentioned above, the presence of SiC_p_ delays the microstructural evolution rate. Therefore, the coarsening of the composite from Ostwald ripening as well as the coalescence of the nearby primary particles is delayed. Secondly, the SiC_p_ located in the liquid phase between the primary particles would hinder the coalescence of the neighboring primary particles and play an obstacle role in atom diffusion. Under these circumstances, the size of the primary particles in the PTF composite is slightly smaller than that in the PTF alloy. In conclusion, the microstructure of the PTF composite differs from that of the PTF alloy. The primary particles are more irregular and the amount of secondarily solidified structures is less than those of the PTF alloy because of the delayed microstructural evolution rate during partial remelting. Besides, the size of the primary particles is slightly smaller than that in the PTF alloy due to the delayed microstructural evolution rate as well as the pinning effect of SiC_p_ against the coalescence of the nearby primary particles.

In an attempt to examine the differences in the eutectics amount of these three materials, the statistical measure was utilized in the Figure 2b,c,f and Figure 3b with the same magnification. Comparison with Figure 2b,c indicates that the eutectics fraction of the PMC alloy is up to 6.7%, which is obviously higher than that of the PTF alloy (3.2%). Nevertheless, comparison with Figure 2f and Figure 3b discloses that the eutectics fraction of the PTF alloy is slightly higher than that of the PTF composite (2.4%). Results from the EDS analysis of the compositions of α-Al phase in these three materials also confirm this viewpoint. As shown in Table 1, the Mg and Si concentrations in these two PTF materials are apparently higher than those in the PMC alloy. During partial remelting, the eutectic phases were dissolving towards the primary α-Al particles, leading to an increase in the Mg and Si concentrations in the primary α-Al particles and a decrease in the liquid. Under these conditions, the formed secondarily primary α-Al grains should increase along with a decrease in the Mg_2_Si based eutectic phases. It is worth noting that the Mg concentration in the PTF composite is slightly lower but the Si concentration is higher than those in the PTF alloy. This is due likely to the reactions of Mg and Al with SiO_2_ on the surfaces of oxidized SiC_p_ at high temperature (above 925 K [18]): 3SiO_2(S)_ + 4Al_(l)_ *→* 2Al_2_O_3(S)_ + 3Si_(S)_; SiO_2(S)_ + 2Mg_(l)_ *→* 2MgO_(S)_ + Si_(S)_ and 2SiO_2(S)_ + 2Al_(l)_ + Mg_(l)_ *→* MgAl_2_O_4(S)_ + 2Si_(S)_

These reactions should reduce Mg concentration but enhance Si concentration in the liquid. Therefore, the diffusion of Mg atom from the liquid to the primary α-Al particles decreased while that of the Si atom increased, resulting in a lower Mg concentration as well as higher Si concentration in the primary α-Al particles of the PTF composite. Since Mg was a vital element to the formation of the eutectics during solidification, the eutectics amount in the PTF composite should be accordingly reduced. Summarily, the amount of Mg_2_Si based eutectic phases in the PTF materials is distinctly less than that in the PMC alloy due to the dissolution of eutectic phases towards the primary α-Al particles during partial remelting. The eutectics amount in PTF composite is slightly less than that in the PTF alloy owing to the reactions of Mg and Al with SiO_2_ on the surfaces of oxidized SiC_p_ as well as the delayed microstructural evolution.

### 3.2. Porosity Evaluation

**Figure 4:**
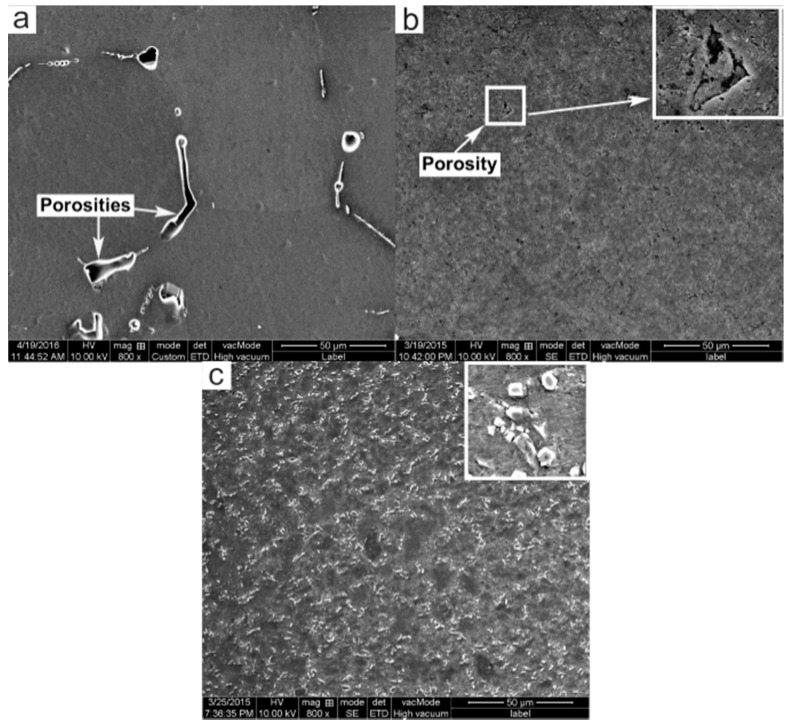
SEM images showing porosities in: (**a**) PMC-6061 alloy; (**b**) PTF-6061 alloy; (**c**) PTF-SiCp/6061Al composite.

Figure 4 shows the typical SEM images of porosity distribution in these three materials under the same magnification. Representative pores can be easily identified in the PMC alloy, as presented in Figure 4a. Nevertheless, the pore amount in the PTF alloy is markedly reduced (Figure 4b) and the PTF composite is actually free of gases and shrinkage porosities (Figure 4c). The porosity percentage of the PTF alloy attains a value of 0.16% and the PTF composite is only 0.07% in comparison with that (3.50%) of the PMC alloy.

The lower porosity of the PTF materials can be attributed to the following factors. First, a high pressure was applied in PTF during solidification, squeezing the liquid metal into the last solidified zone of the casting and consequently diminishing the shrinkage porosities. The ability to feed the solidification shrinkage is thereby improved. Besides, a relatively lower filling velocity was utilized during mold filling to effectively avoid air entrapment. The second one is due to the inherent characteristic of the semisolid forming [19,20,21]. The near-spheroidal morphology of the primary α-Al particles in the PTF materials is more beneficial to liquid penetration for feeding [22]. Moreover, the proper liquid fraction of the PTF, which is lower than that of the PMC, should further decrease the probability of the shrinkage porosities as well as entrapped gases. It should be noted that the compactness of PTF composite is slightly higher than that of the PTF alloy. This is because the SiC_p_ in the secondarily solidified structures may likely fill in these intergranular pores and the pore amount in the PTF composite is therefore lower than that in the PTF alloy.

### 3.3. Tensile Properties

**Figure 5:**
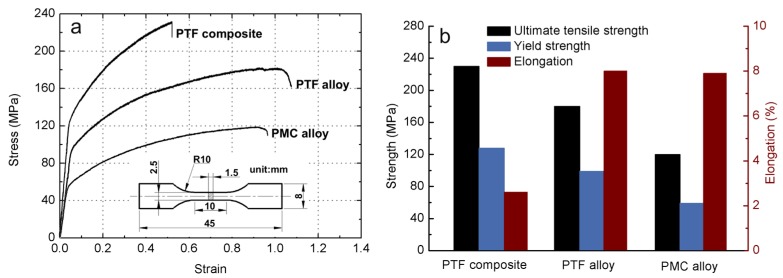
Tensile properties of these three materials: (**a**) stress-strain curves; (**b**) statistical results based on the average of minimum five tests.

Figure 5 presents the mechanical properties of these three materials. It is evident that the ultimate tensile strength and yield strength of the PTF alloy are 180 MPa and 99 MPa, representing an enhancement of 50% and 67.8% respectively in comparison with those (120 MPa and 59 MPa) of the PMC alloy. Besides, the ultimate tensile strength and yield strength of the PTF composite attain the values of 230 MPa and 128 MPa, indicating an increase of 27.8% and 29.3%, respectively, in comparison with those of the PTF alloy. Nevertheless, the elongation of the PTF alloy attains the maximum value of 8.0% among these three materials. That is, the addition of SiC_p_ can significantly enhance the tensile strength but sacrifice the ductility. This is consistent with the results of many investigations on SiC_p_/Al composites [23,24].

The superior tensile properties of the PTF alloy than the PMC alloy can be attributed to the elimination of porosities and the decrease in the average grain size as mentioned above. In addition, the lower amount of Mg_2_Si phase in the PTF alloy should also contribute to its superiority because Mg_2_Si is detrimental to the tensile properties due to its brittle nature [25]. However, the superior tensile properties of the PTF composite than the PTF alloy should be primarily attributed to the existence of the SiC reinforcing particles to strengthen the α-Al phase, and the detailed strengthening mechanisms will be quantitatively discussed in the following sections.

**Figure 6:**
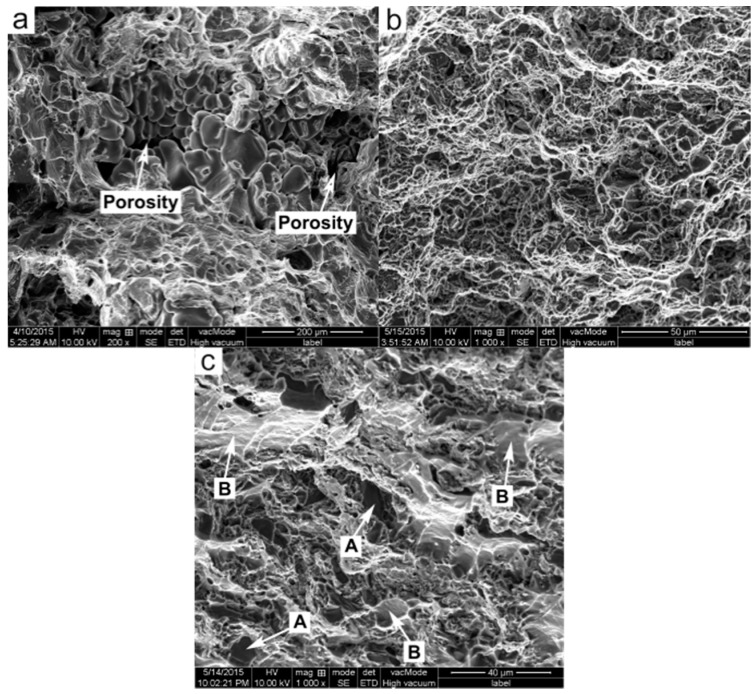
SEM fractographs of: (**a**) PMC-6061 alloy; (**b**) PTF-6061 alloy; (**c**) PTF-SiCp/6061Al composite.

**Figure 7:**
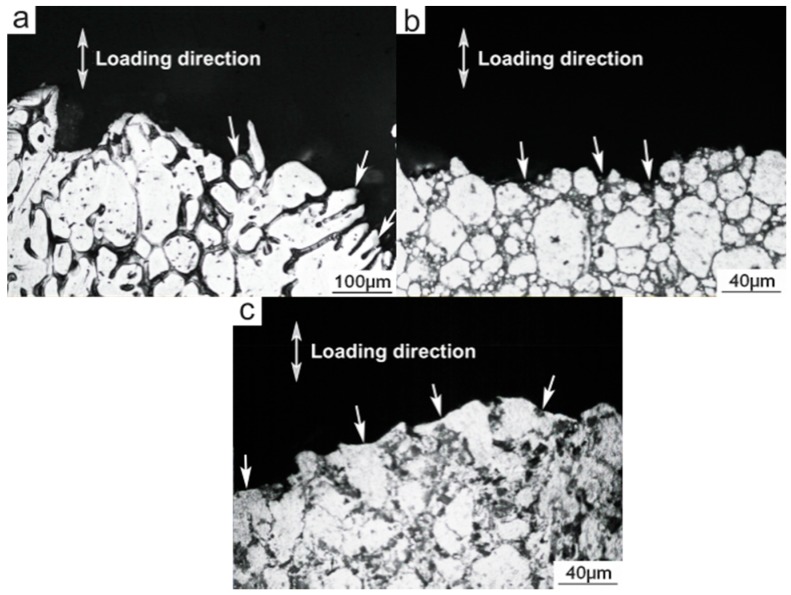
Side views of fracture surfaces of: (**a**) PMC-6061 alloy; (**b**) PTF-6061 alloy; (**c**) PTF-SiCp/6061 Al composite.

Figure 6 presents the typical fractographs of these three materials. The fracture surface of the PMC-6061 alloy is characterized by obvious porosity features (Figure 6a) and thus cracks preferentially originate in (marked by arrows in Figure 7a) and then propagate along the eutectic structures during tensile testing. In general, the fracture of the PMC alloy belongs to intergranular mode. In contrast, the microstructure of the fracture surface of the PTF-6061 alloy is quite compact. Accordingly, the porosity characteristics on the fracture surface of the PTF alloy disappear and are replaced by small dimples (Figure 6b). As mentioned above, thixoforming is effective in reducing or eliminating porosities and gas pores [10,16,19]. It is therefore feasible to give the PTF materials a high density. Differing from the PMC alloy, the fragile eutectic phases in the secondarily solidified structures become the weak points of the PTF alloy and thus cracks preferentially originate in and then develop along these phases in the process of tensile testing (marked by arrows in Figure 7b). Generally, the failure of the PTF alloy still belongs to the intergranular mode.

Figure 6c shows the fracture surface of the PTF-SiC_p_/6061 Al composite, which is characterized by visible SiC_p_ (marked by arrows A) and flat facets (marked by arrows B). The microstructure of the fracture surface, similar to that of the PTF-6061 alloy, retains its compactness. Correspondingly, the dimple characteristics on the fracture surface of the PTF composite disappear and are substituted by flat facets induced by cracks moving through the primary α-Al particles [19]. This accounts for the minimum elongation of the PTF composite in these three materials. The side view of the fracture surface shows that the cracks propagate across the primary α-Al particles (marked by arrows in Figure 7c) and the failure turns into the transgranular fracture mode. This change in the failure mode from intergranular (for the PTF alloy) to transgranular (for the PTF composite) is mainly due to the existence of SiC_p_ in the secondarily solidified structures, which generates a pinning effect against the crack propagation along the secondarily solidified structures during tensile testing. In summary, the fracture surface of the PMC-6061 alloy is characterized by porosity features while that of the PTF-6061 alloy is quite compact. Both of the fractures belong to the intergranular mode. The fracture surface of the PTF-SiCp/6061Al composite is characterized by visible SiC particles and flat facets. The failure turns into the transgranular fracture mode.

### 3.4. Strengthening Mechanisms of Sic_p_

**Figure 8:**
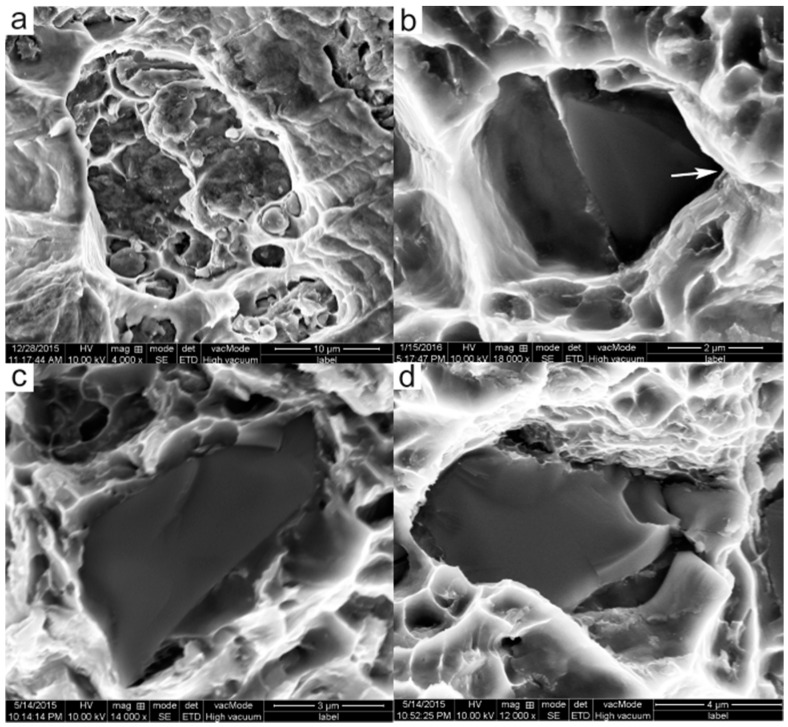
Typical SEM fractographs of the PTF-SiC_p_/6061Al composite. (**a**) fracture across the matrix; (**b**) debonding of the SiC_p_/matrix interface; (**c**,**d**) fragmentation of the SiC_p_.

As mentioned above, the superior tensile strength of the PTF composite should be attributed to the existence of SiC reinforcing particles to strengthen the α-Al phase. In effort to clarify the strengthening mechanisms of SiC_p_ in the PTF-SiC_p_/6061 Al composite, some typical fracture surfaces were observed under high magnification. Results from Figure 8 indicate that there are three kinds of fracture mode in the composites during tensile testing: ductile fracture across the matrix (Figure 8a) induced by the relatively lower matrix strength along with well bonded SiC_p_/matrix interface and two types of failure correlated with SiC_p_. One is the interfacial debonding of SiC_p_/matrix (Figure 8b) and the other is the fragmentation of SiC_p_ (Figure 8c,d). It is thought that the interfaces of SiC_p_/matrix are ascribed to an inherent interface because of the differences in the lattice constants and crystal structures [26]. Under this condition, local stress concentrations generate at the sharp edges and corners of the SiC_p_ in the process of tensile testing (marked by arrow in Figure 8b) and lead to the final debonding of SiC_p_/matrix interfaces. The fragmentation of SiC_p_ includes two ways to fracture: either fractured parallel to the fractured surface (Figure 8c) or broken into several pieces (Figure 8d), which is also induced by the local stress concentrations generated in the SiC_p_/matrix interface.

The possible strengthening mechanisms that may operate in the PTF-SiC_p_/6061Al composite can be mainly divided into two categories: direct and indirect strengthening [27,28]. The direct strengthening, also known as the load transfer model, is based on the load sharing between the reinforcement and the matrix. The indirect strengthening is caused by the changes in the matrix microstructure due to the introduction of reinforcements, and the most used ones for the yield strength prediction of particle reinforced AMCs are Orowan strengthening and CTE mismatch [29,30,31,32]. To get a theoretical evaluation of the yield strength of present composite in the micromechanics approach, a simple linear summation of such terms is utilized [29,33,34]:

(1) $$\sigma =\sigma_{\text{cy}}+\Delta \sigma_{\text{or}}+\Delta \sigma_{\text{CTE}}$$

As is indicated in Figure 8d, the SiC_p_ have split into several parts and there are no obvious cracks in the surrounding matrix, revealing that the load transfer mechanism undoubtedly plays a role in strengthening the matrix. The most used load transfer model is the modified shear lag (MSL) model proposed by Nardon and Prewo [35]:

(2) $$\sigma_{\text{cy}}=\sigma_{\text{my}}\left[\frac{V_{p}\;(S+2)}{2}+V_{m}\right]$$

where σ_cy_ is the composite yield strength, σ_my_ is the matrix yield strength, V_m_ and V_p_ are the volume fractions of matrix and reinforcing particles respectively, S is the average aspect ratio of the reinforcing particles.

For V_p_ + V_m_ = 1, Equation (2) can be substituted by

(3) $$\sigma_{\text{cy}}=\sigma_{\text{my}}\left(1+\frac{V_{p}S}{2}\right)$$

Using the parameters listed in Table 2, Equation (3) can be rewritten as

(4) $$\sigma_{\text{cy}}=99+120.29V_{p}$$

**Figure 9:**
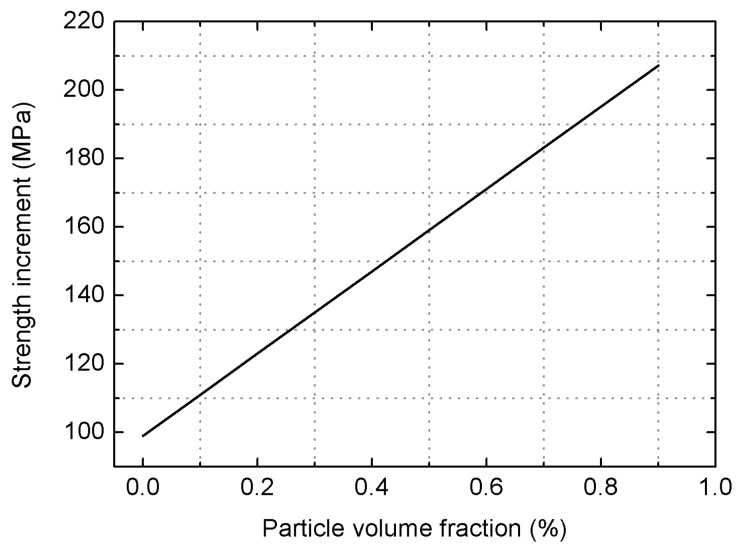
Dependence of the yield strength based on MSL model on the reinforcement volume fraction.

Figure 9 shows a good linear relationship between the yield strength increment and the particle volume fraction. As for the 10 vol% SiC_p_/6061Al composite employed in this work, the increment of yield strength σ_cy_ attains the value of 111 MPa.

The Orowan strengthening is induced by the interactions between the dispersed reinforcing particles and the dislocations due to the second-phase dispersions, precipitates and voids/cavities on the basis of the assumption that the spherical reinforcements ideally distribute uniformly in the matrix. The following expression is often used [37]

(5) $$\Delta \sigma_{\text{or}}=\frac{2\text{Gb}}{0.6d\sqrt{2\pi /V_{p}}}$$

where Δσ_or_ is the yield strength increment brought about by the Orowan strengthening, G is the shear modulus of matrix, b is the Burgers vector and d is the average diameter of reinforcements. Putting the relevant parameters into Equation (5), Δσ_or_ can be expressed in terms of V_p_ by

(6) $$\Delta \sigma_{\text{or}}=1.3738\sqrt{V_{p}}$$

**Figure 10:**
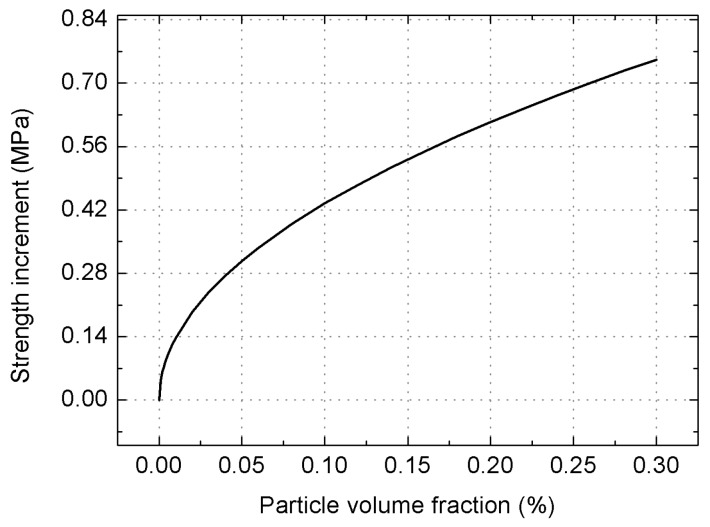
Dependence of the yield strength due to Orowan strengthening on the reinforcement volume fraction.

Figure 10 depicts the variations in the yield strength increments with the particle volume fractions on the basis of Equation (6). For the composite utilized in this work, the increment in yield strength Δσ_or_ assumes the value of only 0.43 MPa.

In addition, the CTE mismatch strengthening is caused by the dislocations resulting from the coefficient of thermal expansion mismatch between the matrix and the ceramic particles [32]. The resultant increment in the yield strength Δσ_CTE_ can be written as

(7) $$\Delta \sigma_{\text{CTE}}=\alpha \text{Gb}\sqrt{\rho }$$

where α is the coefficient of dislocation strengthening and *ρ* is the dislocation density. It follows that

(8) $$\rho =\frac{4V_{p}\Delta T\Delta C}{b\left(1-V_{p}\right)}\left(\frac{1}{t_{1}}+\frac{1}{t_{2}}+\frac{1}{t_{3}}\right)$$

where ΔT is the temperature change from processing temperature to room temperature, ΔC is the CTE difference between matrix and reinforcement particles, and t_1_, t_2_ and t_3_ are the thickness, width and length of reinforcements, respectively. With regard to the SiC_p_ employed in this paper, it was presumed that t_1_ = t_2_ = t_3_ = 6.94 µm. Putting the values of these parameters into Equations (7) and (8), Δσ_CTE_ can be described by V_p_ as

(9) $$\Delta \sigma_{\text{CTE}}=85.61\sqrt{\frac{V_{p}}{1-V_{p}}}$$

**Figure 11:**
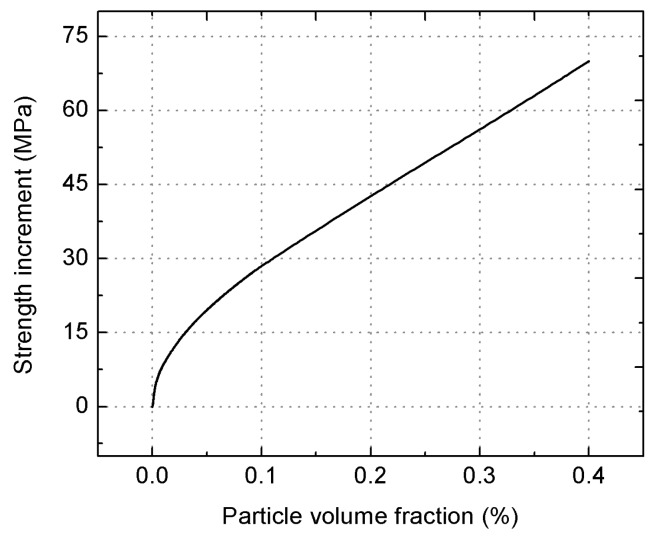
Dependence of the yield strength due to CTE mismatch on the reinforcement volume fraction.

Figure 11 shows the dependence of the yield strength increment on the particle volume fraction described by Equation (9), which shows that the increase in the particle volume fraction markedly enhances the yield strength increment. For the composite studied in this work, the increment in yield strength Δσ_CTE_ retains the value of 28.53 MPa.

From the discussions above, it is found that the direct strengthening due to the load transferring from the matrix to the reinforcements plays the largest role in improving the yield strength. However, the strength increase obtained from the Orowan strengthening is quite minor. This is mainly attributed to the micro size of the employed SiC_p_ and the large inter-particle spacing [12]. Substituting the aforementioned Equations (4), (6) and (9) into Equation (1), it follows that

(10) $$\sigma =99+120.29V_{p}+1.3738\sqrt{V_{p}}+85.61\sqrt{\frac{V_{p}}{1-V_{p}}}$$

The yield strength of the current PTF-SiC_p_/6061Al composite calculated through Equation (10) is 140 MPa, which is apparently higher than the experimental data (128 MPa). This result, as reported by Chen *et al.* [37], can be primarily attributed to the neglect of the influence of the SiC_p_ failure (including SiC_p_/matrix debonding and SiC_p_ cracking) on the composite’s yield strength. As the failed SiC_p_ cannot effectively bear load, a decrease in the yield strength will inevitably appear. Considering these factors, a modified model taking into account the SiC_p_ failure fraction f is proposed

(11) $$\sigma =99+120.29V_{p}\left(1-f\right)+1.3738\sqrt{V_{p}\left(1-f\right)}+85.61\sqrt{\frac{V_{p}\left(1-f\right)}{1-V_{p}\left(1-f\right)}}$$

In accordance with the authors’ previous study [12], the failure fraction f attains the value of 25.6%. Substituting this value into Equation (11), the calculated yield strength for the present composite is 132.6 MPa, which is much closer to the experimental data (128 MPa). Therefore, this model in the form of Equation (11) accurately captures the strengthening mechanisms and discloses the significant effects of SiC_p_ failure including SiC_p_/matrix debonding and SiC_p_ cracking on the yield strength of the PTF-SiC_p_/6061Al composite.

## 4. Conclusions

1. The microstructure of the PMC-6061 alloy is composed of coarse and equiaxed α dendrites with an average size of 90.01 μm and interdendritic net-like eutectic phases, but the microstructure of the PTF-SiC_p_/6061Al composite, similar to that of the PTF-6061 alloy, consists of near-spheroidal primary particles and intergranular secondarily solidified structures except SiC_p_, which are distributed in the secondarily solidified structures.
2. The primary particles of the PTF composite are more irregular and the amount of secondarily solidified structures is lower than those of the PTF alloy because of the delayed microstructural evolution rate during partial remelting. Besides, the size of the primary particles is slightly smaller than that in the PTF alloy due to the delayed microstructural evolution rate as well as the pinning effect of SiC_p_ against the coalescence of the nearby primary particles.
3. The amount of Mg_2_Si based eutectic phases in the PTF materials is distinctly lower than that in the PMC alloy due to the dissolution of eutectic phases towards the primary α-Al particles during partial remelting. The eutectics amount in PTF composite is slightly lower than that in the PTF alloy owing to the reactions of Mg and Al with SiO_2_ on the surfaces of oxidized SiC_p_ as well as the delayed microstructural evolution.
4. The microstructures of the PTF materials are quite compact while that of the PMC alloy is porous. The porosity elimination in the PTF materials is due to the applied high pressure during solidification, the low filling velocity during mold filling, the improved feeding ability of near-spheroidal primary α-Al particles and the low liquid fraction of the semisolid slurry.
5. The ultimate tensile strength and the yield strength of the PTF composite are 230 MPa and 128 MPa, which is apparently higher than those of the PTF (180 MPa and 99 MPa) and PMC (120 MPa and 59 MPa) alloys. The elongation of the PTF alloy attains the maximum value of 8.0% among these three materials.
6. The superior tensile properties of the PTF alloy than the PMC alloy can be attributed to the elimination in porosities and the decrease in the average grain size as well as the lower amount of Mg_2_Si phase in the former alloy. The superior tensile properties of the PTF composite over the PTF alloy should be attributed to the existence of the SiC reinforcing particles to strengthen the α-Al phase.
7. The fracture surface of the PMC-6061 alloy is characterized by porosity features while that of the PTF-6061 alloy is quite compact. Both of the fractures belong to the intergranular mode. The fracture surface of the PTF-SiC_p_/6061Al composite is characterized by visible SiC particles and flat facets. The failure turns into the transgranular fracture mode.
8. The strength increment resulted from MSL model plays largest role in improving the yield strength of the composite. However, the strength increase obtained from the Orowan strengthening is quite minor owing to the micro size of the employed SiC_p_ and the large inter-particle spacing.
9. A modified model taking into consideration the SiC_p_ failure fraction was proposed to calculate the yield strength of the PTF composite. The obtained theoretical results were quite consistent with the experimental data, revealing that the SiC_p_ failure including SiC_p_/matrix debonding and SiC_p_ cracking has a significant effect on the composite’s yield strength.

## Figures and Tables

**Table 1:** Compositions of the primary α-Al particles or the grains of these materials.

Materials	Al (wt%)	Mg (wt%)	Si (wt%)
PMC alloy	99.6	0.3	0.1
PTF alloy	98.5	1.0	0.5
PTF composite	98.0	0.8	1.2

**Table 2:** Parameters employed in the calculations (*m*: matrix; *p*: SiC_p_).

Parameter	Value
*G^m^* [36]	25.6 GPa
*b^m^* [29]	0.28 nm
*S*	2.43
*ΔT*	640 K
*α* [37]	1.4
*C^m^* [29]	23.6 × 10^−6^ K^−1^
*C^p^* [36]	4.3 × 10^−6^ K^−1^
